# The uniqueness of ABCB5 as a full transporter ABCB5FL and a half-transporter-like ABCB5β

**DOI:** 10.20517/cdr.2024.56

**Published:** 2024-08-07

**Authors:** Louise Gerard, Jean-Pierre Gillet

**Affiliations:** Laboratory of Molecular Cancer Biology, URPhyM, NARILIS, University of Namur, Namur 5000, Belgium.

**Keywords:** ABC transporter, half-transporter, multidrug resistance, cancer stem cells

## Abstract

The *ABCB5* gene encodes several isoforms, including two transporters (i.e., ABCB5FL, ABCB5β) and several soluble proteins, such as ABCB5α which has been hypothesized to have a regulatory function. ABCB5FL is a full ABC transporter and is expressed in the testis and prostate, whereas ABCB5β is an atypical half-transporter with a ubiquitous expression pattern. ABCB5β has been shown to mark cancer stem cells in several cancer types. In addition, ABCB5β and ABCB5FL have been shown to play a role in tumorigenesis and multidrug resistance. However, ABCB5β shares its entire protein sequence with ABCB5FL, making them difficult to distinguish. It cannot be excluded that some biological effects described for one transporter may be mediated by the other isoform. Therefore, it is difficult to interpret the available data and some controversies remain regarding their function in cancer cells. In this review, we discuss the data collected on ABCB5 isoforms over the last 20 years and propose a common ground on which we can build further to unravel the pathophysiological roles of ABCB5 transporters.

## INTRODUCTION

ABCB5 was first identified in 1996 and named HuMDR3 after mapping of ABC transporter genes using the human-expressed sequence tags database, which reports cDNA sequences from various tissues representing the human transcriptome^[1]^. This ABC transporter homolog was identified on chromosome 7^[1]^ and first cloned seven years later by Frank *et al*. from human epidermal melanocytes (HEM)^[2]^. This transporter, first named ABCB5 P-gp and later ABCB5β, was shown to influence membrane potential in progenitor cells, which regulated their fusion^[2]^. Soon after, additional transcripts were cloned, including ABCB5α^[3]^, ABCB5.e^[4]^, and ABCB5.ts for testis-specific, also named ABCB5FL for full-length^[5,6]^. According to the AceView program^[7]^, which provides a strictly cDNA-supported view of the human transcriptome and genes, transcription of the *ABCB5* gene results in 11 transcript variants [Table 1].

**Table 1:** Transcript variants of ABCB5

**Given name**	**AceView program**	**Amino acids**	**Ref.**
ABCB5.ts, ABCB5FL (full length)	ABCB5.a	1,257	[5,6]
ABCB5β	ABCB5.b	812	[2,3]
	ABCB5.c	313	
	ABCB5.d	209	
	ABCB5.e	134	[4]
	ABCB5.f	129	
	ABCB5.g	63	
ABCB5α	ABCB5.h	131	[3,4]
	ABCB5.i	126	
	ABCB5.j	46	
	ABCB5.k	17	

The two longest transcripts, ABCB5FL and ABCB5β, are transcribed from two different promoters, as reported in ZENBU, a genome browser for transcriptomic and epigenetic data^[8]^. The other shorter transcripts, resulting from alternative RNA splicing, are most likely derived from ABCB5β transcription, as their tissue localization overlaps. They encode soluble proteins whose function remains to be elucidated.

**Figure 1:**
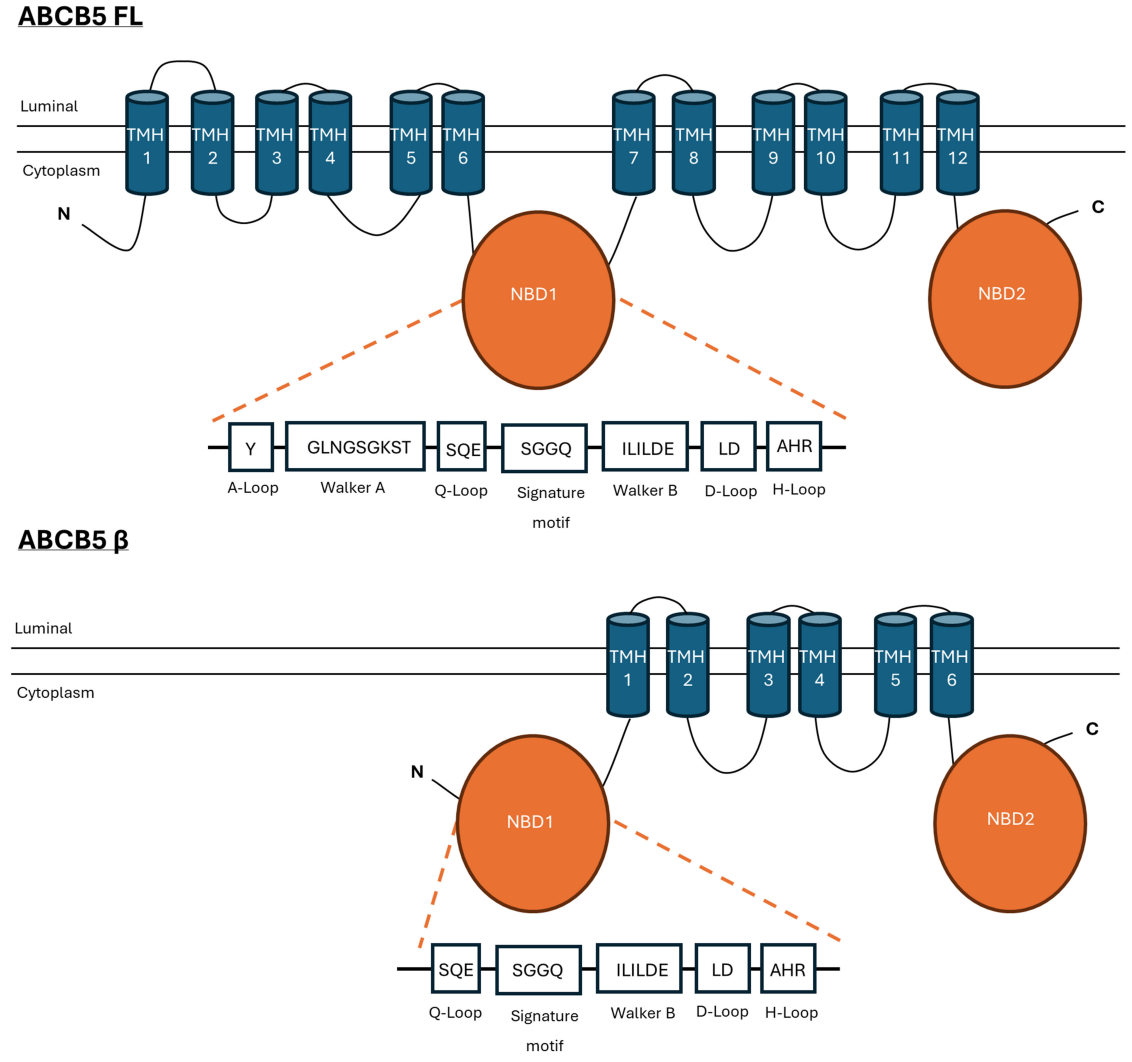
Topology of ABCB5FL and ABCB5β. Two-dimensional representation of the topology of ABCB5FL and ABCB5β based on CCTOP prediction^[9]^. ABCB5FL is composed of 12 TMH (shown in blue) and two NBD (shown in orange). The NBDs are composed of an A-Loop, Walker A, Q-Loop, signature motif, Walker B, D-Loop, and H-Loop; the amino acid sequences are detailed in the corresponding boxes. ABCB5β consists of 6 TMHs and two NBDs. The N-terminal NBD lacks the A-Loop and the Walker A motif. CCTOP: Consensus Constrained TOPology prediction; TMH: transmembrane helice; NBD: nucleotide-binding domain.

ABCB5FL has the typical topology of a full ABC transporter. It is composed of two transmembrane domains (TMDs), each consisting of six transmembrane helices (TMHs), and two nucleotide-binding domains (NBDs) [Figure 1]^[9]^. The TMDs contain the drug binding site, responsible for the interaction with the substrate and are therefore responsible for substrate specificity^[10]^. NBDs are composed of an A-Loop, Walker A, Q-Loop, ABC signature motif, Walker B, D-Loop, and H-Loop [Figure 1]. These conserved motifs are responsible for ATP binding and hydrolysis, interaction between NBDs, and communication between NBDs and TMDs^[10]^. ABCB5β is an atypical half-ABC transporter that harbors an additional NBD in its N-terminus that lacks the A-loop and the Walker A motif involved in ATP binding and hydrolysis [Figure 1]^[11]^. Although ABCB5β was originally predicted to have an extracellular NDB followed by five α-helices and an intracellular NBD^[2]^, two independent studies proposed a more conventional topology consisting of a TMD spanned by six α-helices and two intracellular NBDs^[11,12]^. ABCB5β must either homodimerize or heterodimerize to become functional, and dimerization motifs have been identified in its N-terminal region^[11]^. Recently, our group has shown that the ABCB5β homodimer has a basal ATPase activity that can be inhibited by beryllium fluoride^[12]^. However, the impact of the truncated NBD on the function and expression of the transporter remains to be determined. In this study, we also demonstrated that ABCB5β can heterodimerize with ABCB6 and ABCB9, two other half-ABC transporters, in two melanoma cell lines^[12]^. Chimeric heterodimers showed that ABCB5β/B6 and ABCB5β/B9 have a basal ATPase activity that was reduced by a point mutation, E to Q, in their Walker B motifs^[12]^. This mutation in the Walker B of one transporter or mutations in the Walker B of both interacting partners had the same effect on ATP hydrolysis, suggesting that both transporters are required for ATP hydrolysis^[12]^. It is important to note that even though ABCB5β dimers have basal ATPase activity, there is, so far, no evidence for the physiological relevance of these proteins, and complementary investigations are needed (See Section “ABCB5 physiological function and pathophysiology” for more details).

Overall, ABCB5 isoforms remain little described in the literature and some information is misleading for three main reasons. (1) Conventional techniques (i.e., Western blotting, RT-qPCR, immunofluorescence, and immunohistochemistry) do not always allow to distinguish between the different isoforms; (2) confusion between the two longest isoforms can be seen in many publications; and (3) most of them do not mention the isoform studied. However, ABCB5FL and ABCB5β have different characteristics, as presented in the following section of this review, suggesting that they have different functions. This review proposes to establish common ground by describing seminal information that will help to reconcile the diverse and often disparate literature on ABCB5 transporters.

## EVOLUTION OF THE *ABCB5* GENE

ABCB full transporters are derived from a single gene that is also present in non-vertebrates such as *Drosophila*, *C. elegans,* and yeast^[11]^. In addition to ABCB5, the B family consists of three full transporters (i.e., ABCB1, ABCB4, and ABCB11) and seven half transporters (i.e., ABCB2, ABCB3, ABCB6, ABCB7, ABCB8, ABCB9, and ABCB10). Phylogenetic analysis revealed that ABCB5 is closely related to ABCB1, ABCB4, and ABCB11. ABCB5 homologs have been identified in mice, rats, pigs, chickens, geese, guppies, zebrafish, and earthworms^[13,14]^. ABCB5 shares 73% homology with ABCB1^[15]^. However, in zebrafish, ABCB5 homolog (abcb5) did not transport several ABCB1 substrates, and ABCB4 homolog (abcb4) was shown to have an increased overlapping substrate specificity with ABCB1 than abcb5^[16]^.

The phylogenetic and evolutionary analysis by Moitra *et al*. showed that ABCB5 has evolved as a full transporter for most of its evolutionary history^[11]^. Therefore, it remains puzzling why, at least in humans, ABCB5 also exists as a half-transporter. Ford *et al*. suggested that because ABCB half-transporters are localized in organelles (i.e., mitochondria, lysosomes, and endoplasmic reticulum), it is easier for them to find their interacting partner, thus obviating the need for internal liaison between these proteins^[17]^. On the other hand, full ABCB transporters localize to the plasma membrane. For these transporters, a fusion of the two halves may have occurred to simplify their assembly in response to the increasing complexity of the eukaryotic cell^[17]^. However, this hypothesis is not supported for the ABCG family, where ABCG half-transporters localize to the plasma membrane. Another explanation could be that ABC half-transporters have persisted throughout evolution because they have different interacting partners, with each dimer exerting different functions. Several ABC half-transporters from different families have been shown to interact with each other^[12]^. Although the biological relevance of these interactions remains to be elucidated, it is likely that each dimer has a different function.

## HEALTHY TISSUES AND INTRACELLULAR LOCALIZATION

**Figure 2:**
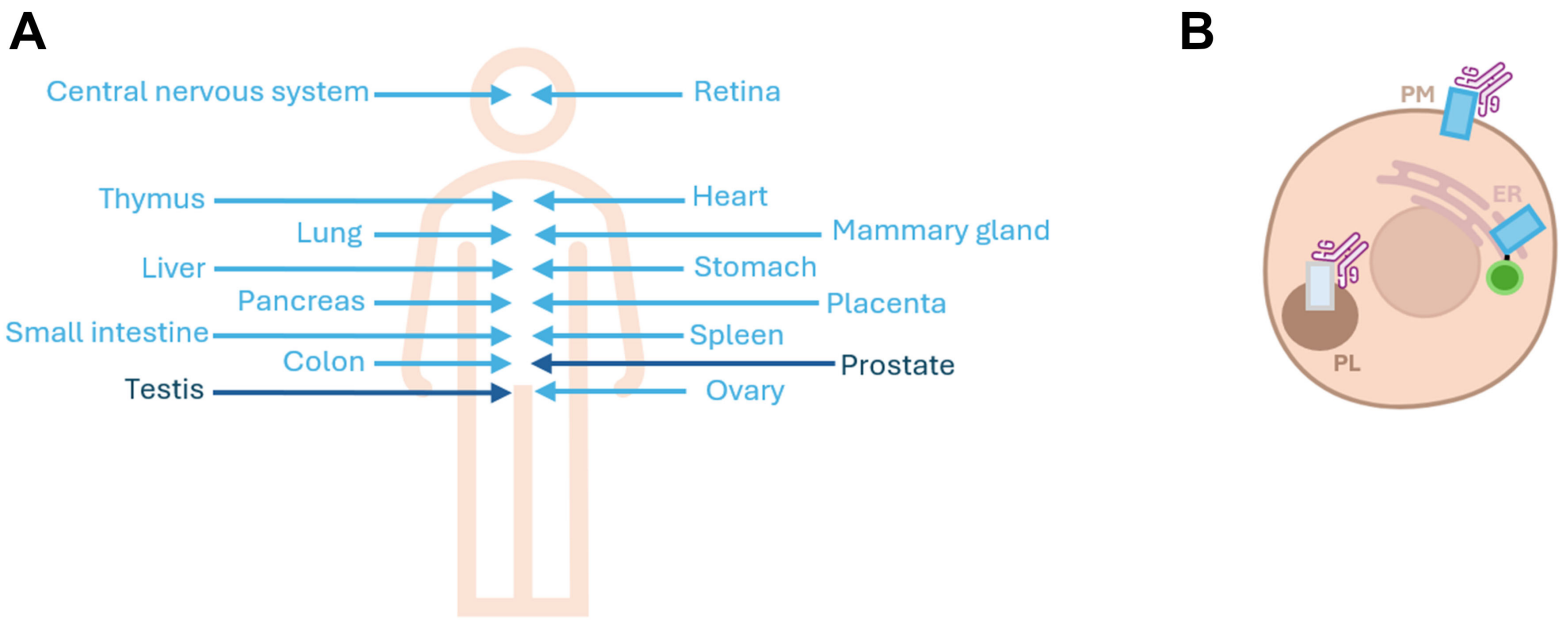
Tissue and intracellular localization of ABCB5FL and ABCB5β. (A) Representation of human ABCB5 tissue localization based on ABCB5FL and ABCB5β mRNA detection from^[1,6,18]^. Light blue represents ABCB5β localization. Dark blue represents ABCB5FL localization; (B) Schematic representation of the proposed intracellular localization of ABCB5β in light blue^[2,19,20]^ and one of the two longest isoforms in gray^[21]^. Results obtained using antibodies, for which more validation is necessary, are highlighted by a pink antibody. Results obtained with tagged protein are highlighted by a green round schematizing a GFP tag. PM: Plasma membrane; ER: endoplasmic reticulum; PL: phagolysosomes; GFP: green fluorescent protein.

ABCB5FL was cloned from a human testis cDNA library, and its expression was detected only in testis and prostate. ABCB5β was cloned from human epidermal melanocyte mRNA but shows a broader tissue expression [Figure 2A]^[2,5,6]^. Saeed *et al*.^[18-21]^ obtained similar results using immunohistochemistry on human tissue^[13]^. They showed that ABCB5β expression is strongest in stalk polyps from the colon, hepatocytes, portal vein, and bile duct, maternal decidua in the placenta and follicles, and thyroid parafollicular cells^[13]^. Furthermore, Jongkhajornpong *et al*. used immunostaining to show that ABCB5 expression in the eye is located in the palisade of Vogt in the basal epithelial layer of the corneal limbus^[22]^. Using immunostaining and *in situ* hybridization, ABCB5 expression in the placenta was observed in villous trophoblasts of first-trimester placentas, partial moles, and complete moles^[23]^. However, to our knowledge, none of these studies used an antibody whose validation has been reported in the literature [Table 2]^[24]^. It is important to emphasize that the main challenge in studying ABCB5 is the specificity of anti-ABCB5 antibodies. Louphrasitthiphol *et al*. were able to decrease ABCB5 mRNA using siRNA, while they were unable to detect a decrease in the levels of the major bands detected by Western blotting using three commercially available anti-ABCB5 antibodies [Table 2, row A]^[25]^. In another study, Díaz-Anaya *et al*. encountered similar problems. A pool of four siRNAs targeting ABCB5 resulted in a greater than 90% decrease in ABCB5 mRNA. However, no decrease in signal was observed by Western blotting and immunofluorescence using three commercially available anti-ABCB5 antibodies [Table 2, row B]^[19]^. Since both studies examined ABCB5 expression 96 h after siRNA transfection, it is very unlikely that the signal in Western blotting is due to residual ABCB5 protein expression. These results call into question the specificity of the antibodies tested and show that we must remain vigilant regarding data published exclusively with anti-ABCB5 antibodies^[19,25]^. Overall, there is a need for standardized methods to validate antibody specificity, and we propose a strategy based on the five pillars proposed by Uhlen *et al*. being genetic validation (using siRNA, shRNA or CRISPR controls), orthogonal validation (comparison with an antibody-independent technique), antibody comparison (comparison with another validated antibody targeting the same protein), validation with a tag (comparison with results obtained using tagged version of the protein of interest), and validation through immunocapture of the target protein followed by mass spectrometry analysis of its expression^[27,28]^. To our knowledge, the lack of validation for anti-ABCB5 is not limited to the antibodies used to investigate ABCB5 localization [Table 2], but it is the same for the remaining literature on this transporter. In consequence, all conclusions published using exclusively antibodies must be carefully taken into account. For the experiments presented in the following Sections, the antibody catalog number and the names of the companies are mentioned to remind the reader of this important limitation.

**Table 2:** Anti-ABCB5 antibodies and their validation

	**Antibody information**	**Epitope ABCB5β**	**Epitope ABCB5FL**	**Validation**	**Concern regarding the localization data**
A	Mouse monoclonal (Catalog number MABC711MI, ThermoFisher Scientific)	aa. 481-674	aa. 926-1119	Western blot with siRNA targeting ABCB5, no decrease in protein level^[25]^	No negative control on the Western blot and no mention of the molecular weight studied. Furthermore, the epitope is localized in the phospholipid bilayer and therefore is not accessible to the antibody by immunohistochemistry^[13]^
	Rabbit polyclonal (Catalog number NBP1-50547x500, Novus Biologicals)	aa. 1-30	aa. 446-475	Western blot comparing protein lysate from Abcb5 wild-type and Abcb5 KO mice. No mention of the molecular weight observed, weak signal for the wild type, and no mention of the tissue of origin of the cells lysed and used for Western blotting^[24]^	The negative control is only an isotype control, i.e., an antibody that has no specificity for the intended target^[22]^. This type of control is used to discriminate signal from background noise, but it does not validate the specificity of the antibody used, as discussed in ref^[26]^. A better control would be the use of knock-out or knock-down cell lines or tissues that do not express the protein of interest
	3C2-1D12 (not commercially available)	aa. 493-508	aa. 938-953	Western blot comparing cells transfected with ABCB5β and non-transfected cells, but no loading control is shown on the Western blot gel^[2]^	No negative control for the immunohistochemistry was performed with the antibody^[23]^
A	Rabbit polyclonal (Catalog number SAB1300315, Sigma)	N-terminus		Western blot with siRNA targeting ABCB5, no decrease in protein level^[25]^	
A	Goat polyclonal (Catalog number ab77549, Abcam)	aa. 460-471	aa. 907-918	Western blot with siRNA targeting ABCB5, no decrease in protein level^[25]^	
B	Rabbit polyclonal (Catalog number ab80108, Abcam)	aa. 1-99	aa. 446-545	Western blot and immunofluorescence after using siRNA targeting ABCB5, no decrease in protein level^[19]^	
B	Rabbit polyclonal (Catalog number 600-401-A77, Rockland)	aa. 192-208	aa. 637-653	Western blot and immunofluorescence after using siRNA targeting ABCB5, no decrease in protein level^[19]^	
B	Rabbit polyclonal (Catalog number Hpa026975, Atlas Antibodies)	aa. 145-234	aa. 590-679	Western blot and immunofluorescence after using siRNA targeting ABCB5, no decrease in protein level^[19]^	

This table shows the antibodies used to detect ABCB5 along with the position of their epitope in the amino acid sequence of both ABCB5β and ABCB5FL isoforms and their proposed validation in the literature. The rows labeled A and B represent the antibodies for which Louphrasitthiphol *et al*. and Díaz-Anaya *et al*. demonstrated a lack of specificity using siRNAs against ABCB5 and Western blotting^[19,25]^. This table purposely presents exclusively antibodies used to study ABCB5 localization or questioned in ref^[19,25]^.

ABCB5 homologs are also ubiquitously expressed in other vertebrates. In zebrafish, abcb5 was detected in liver, kidney, skin, ovary, and gill using a combination of RNAscope and immunohistochemistry^[16]^. Interestingly, abcb5 expression in the ovary was detected in early follicular stages but not in later stages. Similarly, abcb5 expression was detected by *in situ* hybridization in the gills of juvenile rainbow trout, and its expression was restricted to the interlamellar space of the gills, known to contain progenitor cells^[29]^. However, abcb5 expression was not detected in trout liver, kidney, or blood^[29]^. Using immunofluorescence, Saeed *et al*. detected abcb5 expression in the liver of mice, rats, pigs, and chickens^[13]^. In mice, the colon, brain, testes, liver, spleen, kidney, and pancreas had the highest abcb5 expression^[13]^.

Regarding the intracellular localization of ABCB5 [Figure 2B], Frank *et al.* showed,by indirect surface immunostaining and flow cytometry of non-permeabilized MCF-7 transfected with ABCB5β, that ABCB5β was localized to the plasma membrane compared to untransfected MCF-7^[2]^. Although the percentage of ABCB5β expression at the plasma membrane compared to untransfected cells was similar to the transfection rate of this transporter, the Western blotting data used to validate the antibody lacks a loading control necessary to confirm the specificity of this antibody [Table 2]. Subsequently, 14 N-glycosylation sites were found in ABCB5FL, two of which (amino acids 85-88 and 91-94) may be important for plasma membrane targeting^[11]^. Indeed, N-glycosylation has been shown to be necessary for the trafficking of several proteins to the plasma membrane, and the mentioned amino acids are of particular interest due to their position in the ABCB5 sequence^[30]^. In addition, 6 N-glycosylation sites have been found in ABCB5β, one of which (amino acids 374-377) may be important for plasma membrane targeting^[11]^. Chartrain *et al.* also identified ABCB5 expression at the plasma membrane by flow cytometry in several melanoma cell lines^[20]^. They used siRNA against ABCB5 as a negative control and performed their experiment with two different antibodies (600-401-A77 from Rockland and 3C2-1D12 not commercially available) with controversial results in other publications [Table 2]. It is important to note that in this flow cytometry experiment, the cells were not permeabilized, i.e., the antibody used should stain the protein at the cell surface. However, the epitope of 600-401-A77 antibody from Rockland is located at amino acids 192-208 of the ABCB5β sequence (i.e., NBD1), which is thought to be in the cytoplasm, and the epitope of 3C2-1D12 antibody which is not commercially available is located at amino acids 493-508 of the ABCB5β sequence (i.e., the fifth extracellular loop), which is thought to be located outside the cells [Table 2]. Since both epitopes are located on opposite sides of the membrane, it would not have been possible to localize ABCB5β to the plasma membrane by flow cytometry, without permeabilization, by the two antibodies, as suggested by the authors^[20]^. In contrast, Díaz-Anaya *et al*. identified ABCB5β in the endoplasmic reticulum after transfection in HELA and MelJuSo^[19]^. They used a green fluorescent protein (GFP) tag, but several controls were performed to ensure that no mislocalization resulted from the use of this tag. These controls include the localization of GFP-ABCB9, as this transporter localization has already been described in the literature, the use of a smaller tag (HA tag), and the use of a low expression promoter. Cell surface biotinylation in HELA cells showed no expression of labeled ABCB5β at the plasma membrane^[19]^. In another study, ABCB5 was identified by immunofluorescence in phagolysosomes of human monocyte-derived macrophages infected with *Leishmania braziliensis*^[21]^. However, no negative controls were used in this experiment and the reference to the anti-ABCB5 antibody was not mentioned. It is important to point out that the mentioned studies were performed in different cell types and with different techniques, which could be responsible for the different localizations obtained. Overall, the data suggest that ABCB5β localizes in the endoplasmic reticulum, and it is not excluded that after expression in this organelle, ABCB5β traffics to the plasma membrane or to another organelle, although further confirmation on this point is needed. However, our team did not detect its expression at the plasma membrane after cell surface biotinylation in transiently transfected HELA cells. Furthermore, the intracellular localization of ABCB5FL and the other soluble isoforms (including ABCB5α) remains to be elucidated.

## ABCB5 AS A MARKER OF STEM CELLS AND CANCER STEM CELLS

### Stem cells

Stem cells have the unique ability to self-renew and differentiate into any cell of an organism. Interestingly, ABCB5β was first identified as a marker of skin progenitor cells^[2]^. Later, ABCB5 was shown to mark a stem cell subpopulation in several tissues. In the eye, ABCB5 was shown to be a marker of limbal stem cells^[24]^. Ksander *et al*. showed that Abcb5 KO mice have impaired corneal development and corneal restoration after injury^[24]^. They demonstrated that human and mouse ABCB5^+^ limbal stem cells were able to regenerate the cornea in a syngeneic mouse model of limbal stem cell deficiency^[24]^. More recently, this group and others have shown that human ABCB5^+^ limbal stem cells derived from cadaveric corneal tissue can be expanded. Further non-clinical development supported advancement into a clinical trial (NCT03549299) for the treatment of limbal stem cell deficiency^[31]^. Interestingly, Abcb5 KO mice, used to study ABCB5 implication in corneal development, had no visible phenotypical change from litter to adulthood compared to their wild-type counterpart^[24]^. In another study, Abcb5 KO mice treated with haloperidol were shown to have increased brain drug concentration and prolonged haloperidol-induced latency, but no other phenotypes were reported^[32]^.

In human reticular dermis, ABCB5 was also shown to mark a cell subpopulation called dermal immunomodulatory mesenchymal cells, which co-expressed CD29, CD44, CD49e, CD73, CD105, and CD166 stem cell markers^[33]^. This cell subpopulation preferentially expressed programmed cell death protein 1 (PD-1) and was able to modulate primary immune responses in mice after syngeneic or allogeneic *in vivo* injection^[33]^. In a murine heterotopic cardiac allograft model, allogeneic transplantation of ABCB5^+^ dermal immunomodulatory mesenchymal cells increased allograft survival^[33]^. The knockdown of PD-1 in this cell subpopulation attenuated the prolongation of cardiac allograft survival, suggesting that the immunoregulatory function of ABCB5^+^ cells is mediated in part by PD-1 expression^[33]^. ABCB5^+^ dermal immunomodulatory mesenchymal cells have been shown to secrete interleukin-1 receptor antagonist (IL-1RA), which induces a shift from pro-inflammatory M1 macrophages to tissue-healing M2 macrophages^[34]^. Recently, these dermal immunomodulatory mesenchymal cells have been used to enhance tissue repair in patients with chronic venous ulcers refractory to compression therapy^[35,36]^.

### Cancer stem cells

Cancer stem cells (CSCs) are defined as a subpopulation of cells within tumors that are capable of self-renewal, differentiation, and tumorigenicity. These cells have been shown to express ABC transporters, which may explain why CSCs are resistant to chemotherapeutic agents. Recently, it has been proposed that ABC transporters may also be involved in the efflux of molecules (e.g., hormones, signaling molecules, nutrients, metabolites) that are important for intercellular communication or the extracellular environment of CSCs^[37]^.

ABCB5β has been identified as a marker of CSCs in various cancers [Table 3]. However, most experiments in the literature only show the co-expression of several CSC markers with ABCB5, without demonstrating that this enriched ABCB5^+^ cell population has CSC properties. Furthermore, several experiments were performed with antibodies whose specificity was questioned in Table 2.

**Table 3:** Cancer types in which ABCB5 was identified as a marker of CSCs

**Type of cancer**	**Evidence supporting ABCB5 as a marker of CSC**	**Ref.**
Breast cancer	ABCB5 co-expressed with CD133, NANOG, SOX2, and SORT1 in TNBC cells as detected by Western blotting using the antibody 140667 from Abcam	[38]
Colon cancer	CD133^+^ HT-29 cells have increased expression of ABCB5. However, data are not shown and the methodology for this experiment is missing	[39]
	ABCB5 co-expressed with CD133 by immunofluorescence using the antibody 3C2-1D12 which is not commercially available	[40]
Glioblastoma	ABCB5 co-expressed with CD133 in U-87, LN-18 and LN-229 primary tumor cell lines by flow cytometry using antibody 3C2-1D12 which is not commercially available	[41]
Hepatocellular carcinoma	ABCB5 co-expressed with CD133 in hepatocellular carcinoma patient samples by flow cytometry using antibody EB08211 from Everest Biotech Ltd antibody	[42]
	ABCB5 co-expressed with CD133 in Hep3B xenografts by flow cytometry using antibody 600-401-A77 from Rockland	[43]
	Tumorigenicity of purified ABCB5^+^ Hep3B cells by flow cytometry using antibody 600-401-A77 from Rockland and EB08211 from Everest Biotech Ltd antibody was assessed by colony formation assay and injection into BALB/c nude mice. ABCB5^+^ cells had a greater number of colonies compared to ABCB5^-^ cells and mice injected with ABCB5^+^ cells formed tumors in 2 to 4 weeks compared to ABCB5^-^ (12 weeks) and the cell population was heterogeneous	[44]
Mesothelioma	Mesothelioma-initiating cells were selected from human primary samples by culturing the cells in HAM F12/DMEM medium supplemented with 1% PS, 20 ng/mL of EGF, 20 ng/mL of β-FGF, 4 µg/mL of IGF, 0.2% v/v B27, followed by selection by flow cytometry based on Oct4, Nanog, SOX2 and ABCG2 expression. In this subpopulation, ABCB5 expression (mRNA and protein using antibody SAB1300315 from Sigma) was increased	[45]
Oral squamous cell carcinoma	ABCB5 co-expressed with CD44 by immunofluorescence using Hpa026975 antibody from Atlas Antibodies in BICR3 and BICR56 cells	[46]
Osteosarcoma	CSCs of OS-65 cells were distinguished by flow cytometry using Hoechst 33342 dye exclusion. In this SOX2, OCT-4 and NANOG positive cells (confirmed by RT-qPCR and immunofluorescence), ABCB5 mRNA was upregulated by RT-qPCR	[47]
Retinoblastoma	CD133^+^ Y79 cells have an increased expression of ABCB5 mRNA in RT-qPCR compared to CD133^-^ Y79 cells	[48]

Experiments performed to identify ABCB5 as a marker of CSCs are listed as well as the antibody that was used. CSC: Cancer stem cell; TNBC: triple-negative breast cancer; PS: penicillin-streptomycin; EGF: epidermal growth factor; β-FGF: basic fibroblast growth factor; IGF: insulin-like growth factor.

In addition to the cancer types shown in Table 3, ABCB5 has also been shown to mark a CSC population in melanoma. Interestingly, most of the literature investigating the role of ABCB5 as a marker of CSC has focused on this cancer type. First, Frank *et al*. showed that enriched CD133^+^ G3361 cell subpopulation had increased ABCB5 expression^[2]^. ABCB5 marked a distinct cell population from CD133^+^ G3361 melanoma cells, consisting of mononucleated and multinucleated cells. In this experiment, ABCB5 expression was analyzed by flow cytometry using the 3C2-1D12 antibody which is not commercially available. Similarly, ABCB5 mRNA was increased in a human melanoma side population selected by Hoechst 33342 dye exclusion^[49]^. Hoechst dye, which emits a greater amount of fluorescence when bound to DNA, is preferentially excluded from CSCs. In another publication, the number of CD133^+^ ABCB5^+^ cells was higher in advanced-stage human cutaneous melanoma compared to benign nevi^[50]^. However, these data were obtained by immunohistochemistry and the anti-ABCB5 antibody used was not mentioned. Interestingly, by flow cytometry using the 3C2-1D12 antibody, most of the purified CD133^+^ melanoma tumor cells were shown to express ABCB5, and the purified CD133^+^/ABCB5^+^ cells were able to regenerate a heterogeneous tumor in *in vitro* culture^[18]^. ABCB5 and CD133 were also co-expressed in clinical melanoma samples using immunofluorescence and 3C2-1D12 antibody^[18]^. Schatton *et al*. showed that human ABCB5^+^ melanoma cells were able to restore tumor heterogeneity compared to ABCB5^-^ cells when using primary patient-derived tumor cells in human to NOD/SCID mouse xenotransplantation^[51]^. ABCB5^+^ cells were purified using immunomagnetic selection and 3C2-1D12 antibody. Furthermore, in the same publication, ABCB5^+^ cells showed greater tumorigenicity in the G3361 NOD/SCID mouse xenograft model analyzed for 6 weeks^[51]^. ABCB5^+^ melanoma cells purified by immunomagnetic selection and 3C2-1D12 antibody had immunomodulatory functions including inhibition of IL-2 secretion and induction of IL-10 secretion, which affected antitumor immunity by inhibiting T-cell activation^[52]^. In addition, they presented other features involved in immune escape, including decreased major histocompatibility complex type 1 expression and preferential expression of PD-1 and B7.2, two immunotherapy targets. Wilson *et al*. highlighted that in the ABCB5^+^ melanoma cell subpopulation purified by immunomagnetic selection and 3C2-1D12 antibody, ABCB5 is involved in WFDC1 repression (a repressor of the WNT signaling pathway) and IL-1β secretion, which maintains aggressiveness and communication with ABCB5^-^ tumor cells through IL-1β/IL-8/CXCR1 signaling^[53]^. Similarly, the Wnt/IL-1β/IL-8 pathway was shown to induce ABCB5 expression in mesothelioma, and three signaling pathways, NF-κB, α6-β4-integrin, and IL-1, were overexpressed in ABCB5^+^ melanoma cells purified by immunomagnetic selection and abcam 140667 anti-ABCB5 antibody^[54]^. ABCB5 has been shown to activate the NF-κB pathway by inhibiting p65 ubiquitination, thereby increasing its stability.

In contrast, Quintana *et al*. did not observe a correlation between the expression of ABCB5 and 18 other CSC markers using the 3C-1D12 antibody^[55]^. Furthermore, ABCB5^+^ and ABCB5^-^ cells purified by flow cytometry using 3C-1D12 antibody had similar tumorigenic capacities and were able to restore tumor heterogeneity in mouse xenograft models transplanted with Matrigel^[55]^. In melanoma or retinoblastoma cell culture, ABCB5 protein (600-401-A77 antibody from Rockland) or mRNA expression did not correlate with CD271 or CD133 expression^[48,56]^. Consequently, Quintana *et al*. challenged the model of CSCs in melanoma and instead proposed that melanoma is not organized hierarchically, but that each cell undergoes reversible phenotypic changes; this model is called “phenotype switching”^[55]^. Consistent with this, Louphrasitthiphol *et al*., using the TGCA database and the Cancer Cell Line Encyclopedia (CCLE) database, showed that ABCB5 mRNA was associated with MITF mRNA expression, a marker of proliferative/differentiated melanoma cells^[25]^. On the other hand, no correlation was found with AXL, a marker of stemness, suggesting that ABCB5 expression is not exclusive to an undifferentiated stem cell population but rather that this transporter is expressed in differentiated melanoma cells. For a long time, the existence of melanoma cancer stem cells has remained controversial in the literature, and two different models have been proposed, either every cell in a tumor can become tumorigenic due to clonal selection or mutations, or only a subpopulation of cells is tumorigenic, the CSCs^[57]^. Part of the controversy stems from different interpretations of the CSCs concept. CSCs and stem cells share similar characteristics, but they are not further related, and cancer stem cells do not necessarily arise from normal stem cells^[57,58]^. Furthermore, as reviewed by Girouard *et al*., the criteria and methods used to identify and characterize CSCs vary widely in the literature^[57]^. For example, the use of Matrigel may increase tumor formation^[57]^. Although the literature is still controversial regarding the existence of CSCs in melanoma, the “phenotype switching” model is the most commonly used to explain tumorigenesis in recent publications on melanoma formation and progression, which calls into question the expression of ABCB5 in CSCs^[59,60]^. It is important to note that the majority of the literature supporting the role of ABCB5 in CSCs is dominated by the use of the 3C2-1D12 antibody, and when a different antibody is used (in this case 600-401-A77 from Rockland), opposite results are obtained. Further confirmation regarding antibody specificity is needed to disentangle the role played by ABCB5 in CSCs. Moreover, if ABCB5 expression in CSCs happens to be validated, it will be necessary to determine if this transporter is implicated in MDR in this cell type or has another function.

Further, all published data on ABCB5 expression in melanoma CSCs rely on antibodies to isolate ABCB5^+^ cells. These antibodies can recognize at least the two longest isoforms. A similar issue is seen with the probes used to correlate ABCB5 mRNA expression with various CSC markers. Nevertheless, two publications performed side experiments in which ABCB5β can be distinguished, suggesting that ABCB5β is the isoform that is preferentially expressed in melanoma^[2,18]^. A simple confirmation would be to analyze ABCB5FL and ABCB5β mRNA expression in this cell subpopulation using specific probes.

## ABCB5 IN CANCER BIOLOGY AND MULTIDRUG RESISTANCE

High ABCB5 expression has been reported in several malignancies. Using immunofluorescence and MABC711MI antibody from ThermoFisher Scientific, Saeed *et al*. detected ABCB5 expression in various tumors derived from the following tissues (prostate, pancreas, brain, kidney, liver, stomach, ovary, esophagus, pharynx, bladder, and thyroid)^[13]^. ABCB5 expression was the highest in carcinomas of the prostate, pancreas, breast, lung, kidney, liver, and stomach, while the others showed a high interindividual variation of ABCB5 expression. Table 4 highlights the publications in which ABCB5 expression was detected in patient tumor samples^[61-78]^. As shown in Table 4, most of the published data are based on experiments performed exclusively with antibodies and probes. For ABCB5 mRNA expression, the probes always recognize both longest isoforms of ABCB5. A similar problem exists with the antibodies used. Since the probes and antibodies do not distinguish between ABCB5FL and ABCB5β, it is difficult to determine which isoform is predominantly expressed in these cancers. However, based on the expression of ABCB5β in healthy tissues, we can hypothesize that these publications refer to the same isoform. Table 4 only recapitulates the tissue expression of ABCB5, but this transporter was also detected in several cell lines derived from the mentioned tissues. It is interesting to note that most of the melanoma cell lines used in the NCI-60 panel, a group of 60 cancer cell lines used for drug screening, express ABCB5^[3,79,80]^.

**Table 4:** Cancers in which ABCB5 expression has been identified outside of its role as a marker of CSC

**Type of cancer**	**Technique used**	**Ref.**
Breast cancer	RT-qPCR	[61]
	Next-generation sequencing, whole genome sequencing	[62]
Colorectal cancer	Immunohistochemistry using 3C2-1D12 antibody, which is not commercially available	[40]
Hematological malignancies	RT-qPCR	[63]
	RT-qPCR	[64]
	RT-qPCR	[65]
Hepatocellular carcinoma	PCR DNA sequencing	[66]
	RT-qPCR	[67]
	RT-qPCR	[68]
Lung cancer	Next-generation sequencing, whole genome and transcriptome sequencing	[69]
	Next-generation sequencing, whole exome sequencing	[70]
Melanoma	Immunohistochemistry but no information on the antibody used	[71]
	RT-qPCR	[72]
	Immunohistochemistry using antibody ab140667 from Abcam	[73]
	Immunohistochemistry using antibody NBP1-77687 from Novus Biological	[74]
Merkel cell carcinoma	Immunohistochemistry using 3C2-1D12 antibody, which is not commercially available	[75]
Ocular surface squamous neoplasia	Immunohistochemistry using antibody NBP1-50547×500 from Novus Biological	[22]
Oral squamous cell carcinoma	Immunohistochemistry using antibody Hpa026975 from Atlas Antibodies	[76]
	Immunohistochemistry using antibody Hpa026975 from Atlas Antibodies and RT-qPCR	[46]
Pancreatic cancer	RT-qPCR	[77]
Papillary thyroid carcinoma	Immunohistochemistry using antibody GTX60661 from GeneTex	[78]

This table recapitulates the techniques used to identify ABCB5 expression in patient tumors. CSC: Cancer stem cell; RT-qPCR: reverse transcription quantitative polymerase chain reaction.

### Cancer biology

In conjunction with its role as a marker of CSCs, ABCB5 has been associated with cancer progression in several cancer types. Studies have shown (1) a correlation of ABCB5 expression with tumor stage *in vitro* and *in vivo*; (2) a correlation of ABCB5 expression with tumor stage in cancer patient tissues; (3) a correlation of ABCB5 expression with patient survival and tumor progression; and (4) direct *in vitro* and *in vivo* evidence.

(1) ABCB5 expression *in vitro* and *in vivo* was correlated with tumor stage and aggressiveness. Using Western blotting, without specifying the antibody used, and RT-qPCR, it was shown that non-invasive breast cancer cell lines (MDA-MB-468 and MCF7) had decreased expression of ABCB5 compared to an invasive cell line (BT549)^[61]^. Similarly, ABCB5 mRNA was six times less expressed in WM-115, derived from a primary melanoma tumor, compared to WM-266-4, derived from metastasis from the same patient^[20]^. In an orthotopic mouse model of conjunctival melanoma, ABCB5 expression, analyzed by flow cytometry using the 3C2-1D12 antibody which is not commercially available, increased during tumor expansion phases and its expression was higher in metastasis^[81]^.

(2) ABCB5 expression has been shown to be higher in more advanced stages of cancer, metastasis, and refractory tumors when examined by immunohistochemistry or RT-qPCR in patients with colorectal cancer, oral squamous cell carcinoma, leukemia, and breast cancer^[40,46,61,63]^. In immunohistochemistry, ABCB5 expression gradually increases from benign nevus to invasive melanoma and from normal oral mucosal tissue to oral precancerous lesions and squamous intraepithelial neoplasia^[71,73,76]^. Transcriptomic data from different datasets showed that ABCB5 is overexpressed in alveolar soft-part sarcoma^[82]^. ABCB5 expression was increased in ocular surface squamous cell carcinoma compared to normal limbal tissue by immunohistochemistry^[22]^. Furthermore, its expression was no longer restricted to the basal epithelial layer but reached suprabasal and superficial cells in tumor tissue. However, tumor recurrence in ocular surface squamous cell carcinoma after treatment is rare, and when it does occur, re-administration of the same anticancer agent is often effective^[22]^. Because of the ABCB5 expression pattern in tumors of cancer patients and because multidrug resistance is rarely seen in ocular surface squamous neoplasia where ABCB5 is overexpressed, the hypothesis that ABCB5 may be involved in tumor progression is strengthened.

(3) ABCB5 expression has been shown to correlate with overall survival and/or tumor progression in several cancer types, as summarized in Table 5^[83-86]^. RT-qPCR identified ABCB5 as a marker of circulating melanoma cells in the peripheral blood of patients and its expression correlated with disease progression and recurrence^[86,87]^. ABCB5 mRNA was upregulated in the peripheral blood of colorectal cancer patients and its expression correlated with cancer progression^[84]^. Similarly, ABCB5 mRNA in the bone marrow of colorectal cancer patients was negatively associated with tumor progression and overall survival^[83]^. Grimm *et al*. showed that ABCB5 expression, analyzed by immunohistochemistry, was associated with tumor progression and recurrence in patients with oral squamous cell carcinoma^[46]^. Other studies have shown that ABCB5 expression, by immunochemistry, is associated with tumor thickness, a prognostic factor for patient survival^[74,78]^. Whole genome sequencing has shown that various single nucleotide polymorphisms (SNPs), i.e., single nucleotide variations in the DNA sequence that occur in more than 1% of the population, of ABCB5 were associated with cancer occurrence in sarcoma and hepatocellular carcinoma (rs2074000, rs58795451, rs751879475, rs73684574, rs78879263, rs78155891, rs75494098, rs4721940, rs10254317)^[66,88]^. In hepatocellular carcinoma, SNPs were also associated with tumor size (rs73076550, rs75494098, rs76859629, rs12669250), tumor stage (rs2106562, rs17143187, rs17143212, rs2074000, rs10254317), and disease-free survival (rs2893006, rs34603556, ss836312078, rs79998607, ss1148219560, rs111872870, rs75494098, rs76859629, rs11769236, rs11772926, ss836312077 and ss836312079)^[66]^. Using data from two gene expression databases, The Cancer Genome Atlas Program (TCGA) and Gene Expression Omnibus (GEO), Shang *et al*. showed that ABCB5 expression in gastric cancer was associated with increased metastasis and worse overall survival^[85]^. Furthermore, sequencing showed that SNPs of ABCB5 were associated with decreased melanoma risk (i.e., rs10231520, rs17817117, and rs2301641)^[89]^. The rs2301641 SNP encodes a nonsynonymous mutation, K115E, which is associated with lower melanoma risk and reduced ABCB5β transport capacity. In addition, a missense substitution in the melanocyte-inducing transcription factor (MITF), E318K, has been associated with a predisposition to melanoma and renal cell carcinoma^[90]^. MITF has previously been shown to bind to the ABCB5 promoter region and regulate its expression^[25]^. ABCB5-mediated transcription by MITF was induced by β-catenin^[25]^. The E318K mutation increases the binding of MITF to the ABCB5 locus, resulting in the upregulation of ABCB5^[90]^. In the same theme, several transcription factors have been shown to influence ABCB5 expression. The proto-oncogene c-Myc binds to the ABCB5 promoter region and increases its expression^[39,91]^. The p73 isoform ΔNp73, a member of the p53 family, was able to regulate ABCB5 expression in breast cancer cells^[92]^. In addition, several microRNAs (miRNAs) were shown to decrease ABCB5 expression. miRNAs are single-stranded non-coding RNA that prevent mRNA translation into protein. miR-4282 and miR-522 were shown to bind to the ABCB5 sequence in pancreatic and colon cancer cell lines, respectively, using luciferase assays^[77,93]^. Overexpression of these miRNAs negatively affected either the resistance of cancer cells or their ability to migrate. Taken together, they represent a promising tool for cancer research and targeting of ABCB5. Nevertheless, further investigations are needed to better characterize the mechanisms and underlying effect of these miRNAs in colon and pancreatic cancer, but also in other malignancies.

**Table 5:** Association between ABCB5 expression in patients’ tumor and clinical outcome

**Tumor**	**ABCB5 expression**	**Clinical outcomes**	**Ref.**
Colorectal adenocarcinoma	RT-qPCR on ABCB5 mRNA in patient bone marrow	→ Increased tumor progression → Increased tumor recurrence → Decreased overall survival	[83]
Colorectal adenocarcinoma	RT-qPCR on ABCB5 mRNA in circulating tumor cells of peripheral blood from patients	→ Increased tumor progression → Decreased overall survival	[84]
Gastric cancer	ABCB5 mRNA from TCGA and GEO database	→ Poor prognosis	[85]
Melanoma	RT-qPCR on ABCB5 mRNA in circulating tumor cells of peripheral blood from patients	→ Increased tumor recurrence	[86]
Oral squamous cell carcinoma	Immunohistochemistry of ABCB5 expression in tumor samples	→ Increased tumor recurrence → Increased tumor progression	[46]
Papillary thyroid cancer	Immunohistochemistry of ABCB5 expression in tumor samples	→ Increased tumor size	[78]

Patients’ tumors where ABCB5 expression has been associated with clinical outcomes are presented. GEO: Gene Expression Omnibus.

(4) It is important to emphasize that the experiments mentioned in (1), (2), and (3) provide only correlation data. To accurately interpret these findings, they must be supported by experimental evidence including downregulation and overexpression experiments. For instance, Yao *et al*. showed that decreased ABCB5 expression after shRNA reduced cell migration and invasion in Transwell migration and Matrigel invasion assays in a breast cancer cell line^[61]^. In addition, shRNA against ABCB5 in these cells resulted in fewer metastases after tail vein injection of mice. On the other hand, overexpression of ABCB5β resulted in increased migration and invasion *in vitro* and metastasis *in vivo*^[61]^. siRNA against the zinc finger E-Box binding homeobox 1 (ZEB1) oncogene had similar results to ABCB5 knockdown^[61]^. The authors suggested that ZEB1 expression is regulated by ABCB5, but the published data using luciferase assay and qChIP assay suggest that ABCB5 acts as a transcription factor and binds to the ZEB1 promoter region to regulate its expression, an unconventional function for a transmembrane protein. In a xenograft mouse model or *in vitro* culture of colorectal cancer cells, ABCB5 was shown to promote invasion and epithelial-to-mesenchymal transition (EMT)^[84]^. Invasion and EMT were mediated by IL-8 induction of the oncogene receptor tyrosine kinase AXL and were dependent on ABCB5 expression^[84]^. All these experimental data suggest that ABCB5 functions as an oncogene. Conversely, Govindan et al. detected three nonsynonymous mutations by sequencing in 17 patients with non-small cell lung cancer^[69]^. In various cancers, 134 missense mutations and 20 truncating mutations were detected in ABCB5^[94]^. Furthermore, Sana *et al*. showed that ABCB5FL was mutated in 13.75% of the 640 human melanoma samples tested^[95]^. ATPase assays showed that these mutations resulted in decreased ATP hydrolysis and increased proliferation, migration, and invasion. The effects were greater in the melanoma cell lines carrying the NRAS activating mutation, Q16K, except for invasion capacity, which was only seen in cell lines carrying the BRAF activating mutation, V600E^[95]^. To validate these data, the authors used a shRNA targeting ABCB5 in A375 and SK-Mel-28. Downregulation of ABCB5 resulted in increased proliferation of SK-Mel-28, and larger colonies for both cell lines in an anchorage-independent soft agar assay^[95]^. Since ABCB5β is the isoform constitutively expressed in these cell types, the identified phenotype cannot be attributed to the downregulation of ABCB5FL, but rather to the decreased expression of ABCB5β. However, it suggests that ABCB5β functions as a tumor suppressor in the opposite direction to the data presented above. The conflicting results observed may be because the two longest isoforms may have opposite functions in cancer biology. To date, data suggest that ABCB5FL acts as a tumor suppressor while ABCB5β is an oncogene. However, the existence of conflicting results on the same isoform raises questions and further experiments are needed to better determine the implication of each isoform in cancer biology. Additionally, we must keep in mind that shRNA experiments have limitations including off-target effects and limited efficacy. In both publications using shRNA, the downregulation of ABCB5 is confirmed by looking at its mRNA expression and/or protein level using an antibody that was shown to lack specificity^[61,95]^. Moreover, since antibodies do not allow to distinguish between these two proteins, experiments need to be properly designed and the addition of a rescue experiment with transfection of ABCB5β and ABCB5FL sequences separately might help to better identify the transporter responsible for the observed phenotype.

### Multidrug resistance

ABCB1 has been extensively studied for its role in multidrug resistance through the efflux of chemotherapeutic drugs from cancer cells, leading to treatment failure. Due to their high homology, ABCB5 was expected to have a similar function, and this has been reported in several publications. Regarding the involvement of ABCB5 in cancer biology, different types of evidence can be found in the literature: (1) correlation of ABCB5 expression and treatment with anticancer agents; (2) sensitization of cancer cell lines after siRNA or shRNA targeting ABCB5; (3) experimental data studying each isoform separately. Data showing an implication for ABCB5 in the transport of anticancer agents are summarized in Table 6 and divided into direct and indirect evidence of transport.

**Table 6:** List of anticancer agents to which ABCB5 has been proposed to mediate resistance

**Anticancer agent**	**Direct evidence that ABCB5 transports anticancer agent**	**Indirect evidence that ABCB5 transports anticancer agent**
Adriamycin		Increased ABCB5 mRNA after Adriamycin treatment in breast cancer patients^[96]^
Camptothecin	SiRNA against ABCB5 resensitized SK-Mel 28 to camptothecin^[97]^	
Carboplatin		Increased ABCB5 mRNA after selection with carboplatin of MKL-1, MKL-2, MS-1, and WaGa cells^[75]^
		Increased ABCB5 mRNA after treatment with carboplatin in patients with Merkel cell carcinoma^[75]^
Dacarbazine	shRNA against ABCB5 or anti-ABCB5 mAb blockade of ABCB5 on G3361 melanoma cells resulted in decreased survival^[53]^	Increased ABCB5 mRNA after dacarbazine treatment in melanoma patients^[20]^
		Treatment of WM-266-4 cells with dacarbazine results in selection of ABCB5-expressing cells^[20]^
Docetaxel	shRNA against ABCB5 or anti-ABCB5 mAb blockade of ABCB5 on G3361 melanoma cells resulted in decreased survival^[53]^	
	HEK-293 transfected with ABCB5FL were more resistant to docetaxel and showed decreased uptake of this anticancer agent^[6]^	
	Stimulation of ABCB5FL ATPase activity in the presence of docetaxel^[6]^	
Doxorubicin	shRNA against ABCB5 or anti-ABCB5 mAb blockade of ABCB5 on G3361 melanoma cells resulted in decreased survival^[53]^	Increased ABCB5 mRNA after doxorubicin selection of MCF-7 cell^[98]^
	G3361 melanoma cells expressing ABCB5 accumulate less doxorubicin^[75]^	Increased ABCB5 mRNA after doxorubicin treatment of A375, 1205Lu, and DMBC8^[99]^
	Anti-ABCB5 mAb blockade of ABCB5 on G3361 melanoma cells increases doxorubicin uptake^[75]^	Increased ABCB5 mRNA after treatment with doxorubicin in breast cancer patients^[96]^
	Liver cancer cells overexpressing ABCB5 have a decreased doxorubicin uptake^[68]^	
	SiRNA against ABCB5 sensitized liver cancer cells to doxorubicin^[68]^	
Epirubicin		Increased ABCB5 mRNA after treatment with epirubicin in breast cancer patients^[96]^
Etoposide	shRNA against ABCB5 or anti-ABCB5 mAb blockade of ABCB5 on G3361 melanoma cells resulted in decreased survival^[53]^	Increased ABCB5 mRNA after etoposide selection of MKL-1, MKL-2, MS-1, and WaGa cells^[75]^
		Increased ABCB5 mRNA after treatment with carboplatin in patients with Merkel cell carcinoma^[75]^
Paclitaxel	shRNA against ABCB5 or anti-ABCB5 mAb blockade of ABCB5 on G3361 melanoma cells resulted in decreased survival^[53]^	
	HEK-293 transfected with ABCB5FL were more resistant to paclitaxel and showed decreased uptake of this anticancer agent^[6]^	
Temozolomide	shRNA against ABCB5 or anti-ABCB5 mAb blockade of ABCB5 resulted in decreased proliferation of glioblastoma multiforme and sensitized cells to temozolomide in both cell culture and xenograft models^[41]^	Treatment of xenografts in mice with temozolomide resulted in the selection of ABCB5-expressing cells^[20]^
Teniposide	shRNA against ABCB5 or anti-ABCB5 mAb blockade of ABCB5 on G3361 melanoma cells resulted in decreased survival^[53]^	
Vemurafenib		Treatment of WM-266-4 cells with vemurafenib resulted in the selection of ABCB5-expressing cells^[20]^
		Increased ABCB5 mRNA and protein expression in cell lines resistant to vemurafenib^[100]^
Vinblastine		Increased ABCB5 mRNA after vinblastine selection of K562 cells^[101]^
Vincristine	shRNA against ABCB5 or anti-ABCB5 mAb blockade of ABCB5 in G3361 melanoma cells resulted in decreased survival^[53]^	
5-FU	SiRNA against ABCB5 resensitized SK-Mel 28 to 5-FU^[97]^	Treatment of colorectal cancer patients with 5-FU increased ABCB5 protein expression in their tumors^[40]^
	ShRNA against ABCB5 resensitized colorectal cancer xenografts to 5-FU^[40]^	
10-OH camptothecin	SiRNA against ABCB5 resensitized SK-Mel 28 to 10-OH camptothecin^[97]^	

Anticancer agents and the experiments suggesting ABCB5 transport of these drugs are presented in this table. The experiments are divided into two categories: (1) direct evidence and (2) indirect evidence.

(1) ABCB5 mRNA was increased in MCF-7 and K562 cells selected with doxorubicin or vinblastine, respectively^[98,101]^. Treatment with 5 µM doxorubicin resulted in increased ABCB5 mRNA expression in three melanoma cell lines (i.e., A375, 1205Lu, and DMBC8)^[99]^. Selection of MKL-1, MKL-2, MS-1, and WaGa cells with carboplatin and etoposide resulted in increased expression of ABCB5 mRNA^[75]^. Patients with resistant acute myeloid leukemia, breast cancer, and Merkel cell carcinoma had increased expression of ABCB5 mRNA after standard treatment with chemotherapeutic agents^[64,75,96]^. Similarly, patients with melanoma treated with dacarbazine had increased ABCB5 mRNA expression, and treatment with dacarbazine *in vitro* resulted in the selection of ABCB5-expressing cells^[20]^. Colorectal cancer patients treated with 5-FU had increased ABCB5 expression on immunohistochemistry compared to the same tissue before treatment^[40]^. In breast cancer patients, the ABCB5 SNP rs3210441 was associated with response to neoadjuvant cytotoxic therapy and ABCB5 expression in TCGA was associated with anthracycline resistance^[62,96]^. In a murine xenograft model of melanoma, temozolomide led to the selection of ABCB5-expressing cells^[20]^. In addition, abcb5 overexpression in zebrafish reduced mercury toxicity, and in mice, abcb5 expression at the blood-brain barrier was shown to influence brain levels of haloperidol^[32,102]^. Notably, ABCB5 expression at mRNA and protein levels was increased in cell lines resistant to vemurafenib, a molecule targeting the BRAF-activating mutation V600E used in the treatment of melanoma^[100]^. However, ABCB5 was not responsible for resistance to vemurafenib.

(2) Once again, it is important to emphasize that the experiments mentioned in (1) only provide correlation data. To interpret these findings correctly, they must be supported by additional experimental evidence, such as downregulation and overexpression studies. For instance, SiRNA against ABCB5 has been shown to sensitize Sk-Mel 28 cells to camptothecin and 5-FU^[97]^. Similarly, shRNA targeting ABCB5 inhibited cancer growth and sensitized cells to 5-FU in a colorectal cancer xenograft^[40]^. Blockade of ABCB5 with an anti-ABCB5 mAb reduced cancer growth in a Merkel cell carcinoma xenograft^[75]^. G3361 melanoma cells expressing ABCB5 accumulated less doxorubicin and blockade of ABCB5 with the same anti-ABCB5 mAb increased doxorubicin uptake^[18]^. However, both studies use an anti-ABCB5 mAb (3C2-1D12) for which there is no experimental evidence of ABCB5 inhibition. The antibody recognizes amino acids in one of the extracellular loops of ABCB5, on the opposite side of the membrane from the NDBs. Therefore, the inhibition of this transporter is not mediated by ATP hydrolysis blocking. Furthermore, there is no evidence that this antibody interferes with substrate binding and both publications do not confirm the results obtained with ABCB5 downregulation using shRNA. Nevertheless, further experiments in the literature confirmed their results using ABCB5 downregulation. Liver cancer cells overexpressing ABCB5 had decreased uptake of doxorubicin and siRNA against ABCB5 sensitized cells to doxorubicin^[68]^. shRNA against ABCB5 or anti-ABCB5 mAb on G3361 melanoma cells decreased cancer cell survival to dacarbazine, paclitaxel, teniposide, docetaxel, etoposide, doxorubicin, and vincristine^[53]^. Similarly, blockade of ABCB5 with anti-ABCB5 mAb or downregulation with shRNA resulted in decreased proliferation of glioblastoma multiforme and sensitized cells to temozolomide in both cell culture and xenograft models^[41]^. In addition, ABCB5 was shown to mediate resistance to caffeic acid phenethyl ester, a bioactive molecule with antitumor activity, in melanoma^[103]^. However, as mentioned above, we must keep in mind that siRNA and shRNA experiments have limitations including off-target effects and limited efficacy. Further, several publications lack proper proof of validation of ABCB5 downregulation in addition to using antibodies not validated in the literature^[40,53]^.

(3) In contrast to other publications that did not study a specific isoform, Keniya *et al*. decided to study the role of both isoforms separately in chemoresistance^[15]^. In a yeast model, *Saccharomyces cerevisiae*, overexpression of ABCB5FL and ABCB1 mediated resistance to rhodamine 123 whereas ABCB5β did not^[15]^. Conversely, Frank *et al*. showed that ABCB5β was able to transport rhodamine 123 in transfected MCF-7 cells^[2]^. This could be explained by the fact that yeast lacks some human post-translational modifications and/or ABCB5β interacting partners that could affect ABCB5β transporter function, leading to opposite results in the two tested models. Nevertheless, Kawanobe *et al*. reported that HEK-293 cells overexpressing ABCB5FL were 1.5-fold more resistant to doxorubicin, 2.3-fold more resistant to paclitaxel, 3.0-fold more resistant to docetaxel, and 1.2-fold to 1.5-fold more resistant to daunorubicin, vincristine, etoposide, and actinomycin D than the parental HEK-293 cell line^[6]^. These results were obtained in two different clones with the highest ABCB5FL expression out of seventeen and no resistance was observed in the mixed population of clones.

The fold change obtained for doxorubicin, daunorubicin, vincristine, etoposide, and actinomycin D is low and this experiment could have benefited from other validation. Regarding docetaxel and paclitaxel, HEK-293 cells overexpressing ABCB5FL had decreased uptake of radiolabeled drug. Resistance to methotrexate and 5-fluorouracil was not observed^[6]^. In addition, when expressed in Sf21 insect cells, ABCB5FL and ABCB1 ATPase activity were 1.25-fold and 1.11-fold higher, respectively, in the presence of 100 µM docetaxel^[6]^. However, the observed fold change in ATPase activity is quite low and the concentration of docetaxel is particularly high compared to similar assays performed on other ABC transporters, which could affect membrane integrity. Overall, confirmations are needed to determine the implication of ABCB5FL in the transport of anticancer agents. Notably, the only difference between the two isoforms is the absence of the first TMD of ABCB5FL, the A-Loop, and Walker A in ABCB5β. However, using molecular docking, Tangella *et al*. showed that the drug-binding pocket of several ABCB5 substrates (i.e., doxorubicin, daunorubicin, paclitaxel, vincristine, camptothecin, etoposide, docetaxel, and mitoxantrone) is located in the TMH present in TMD1 and TMD2 of ABCB5FL^[104]^. Three potential binding sites were identified: site-1 (including residues in TMHs 1, 3, 4, 5, 6, 7, 8, 9, 10, 11, and 12), site-2 (including residues in TMHs 4, 5, 7, 8, 9 and 10), and site-3 (including residues in TMHs 2, 3, 6, 10 and 11). Therefore, ABCB5β may not be able to transport these anticancer agents because this transporter lacks residues involved in substrate binding that are expressed in ABCB5FL. However, it is not excluded that both transporters transport chemotherapeutic agents. A larger number of compounds needs to be tested in different models to better distinguish which isoform is responsible for the observed multidrug resistance phenotype. Furthermore, the literature lacks transport assays in vesicles with radiolabeled or fluorescent drugs for these transporters. Interestingly, the putative binding sites discovered in ABCB5FL through molecular docking overlap with three binding sites found in human ABCB1 (i.e., prazosin-sites, H-sites and R-sites)^[104]^. Therefore, ABCB5FL was hypothesized to have a substrate spectrum similar to ABCB1. However, in Sf21 insect cell membrane vesicles, ABCB5FL ATPase activity was not stimulated by verapamil, a known substrate of ABCB1 used to inhibit its transport function^[6]^. On the other hand, pretreatment of WM35 melanoma cells, expressing ABCB5 but not ABCB1, with verapamil resulted in increased intracellular concentration of doxorubicin, suggesting the inhibition of this transporter by verapamil^[105]^. However, further experimental evidence is needed to confirm the inhibition and the impact on doxorubicin transport. Additionally, verapamil stimulated ATPase activity of Abcb5 in zebrafish and this stimulation was inhibited by tariquidar^[16]^. Further, incubation of zebrafish with tariquidar led to a slight increase of Abcb5 mRNA, which was not significant^[106]^. Overall, it is not clear whether ABCB1 inhibitors are also potent inhibitors of ABCB5 and transport assay with fluorescent or radiolabeled molecules will help identify ABCB5 inhibitors.

## ABCB5 PHYSIOLOGICAL FUNCTION AND PATHOPHYSIOLOGY

So far, the physiological function of all ABCB5 isoforms remains unknown. Even though the literature lacks evidence that these proteins are physiologically relevant, phenotype changes seen after overexpression or knockout of ABCB5FL or ABCB5β, respectively, have led to relevant hypotheses that must be further verified. HEK-293 cells overexpressing ABCB5FL were resistant to buthionine sulfoximine (BSO), an inhibitor of glutathione synthesis, resulting in increased glutathione levels^[107]^. However, BSO uptake levels were similar in HEK-293 overexpressing ABCB5FL compared to the negative control. It was later discovered that HEK-293 cells overexpressing ABCB5FL also had increased levels of STAT1 and glutaminase, which were responsible for the observed resistance^[108]^. However, STAT1 and glutaminase were not overexpressed or downregulated after transient transfection or siRNA targeting ABCB5; their increased expression was only detectable after stable transfection of this transporter. Therefore, metabolomic changes may occur after ABCB5FL overexpression that affect STAT1 expression over time. This hypothesis was confirmed by the analysis of the amino acid content of the ABCB5FL overexpressing HEK-293 cells and their WT counterparts. HEK-293 cells overexpressing ABCB5FL had increased cellular levels of glutamic acid, aspartic acid, and alanine, and decreased cellular levels of phenylalanine, tryptophan, leucine, isoleucine, glycine, methionine, valine, histidine, and tyrosine. Notably, STAT1 and glutaminase are involved in glutathione synthesis. The latter is an antioxidant involved in cellular detoxification and may be related to the CSC phenotype observed in cells overexpressing ABCB5. However, evidence to date suggests that ABCB5β, and not ABCB5FL, is the isoform expressed in CSC. Since ABCB5FL localization is restricted to the testis and prostate, it has been proposed that it transports androgens^[6]^. However, androgens did not affect the ATPase activity of ABCB5FL in ATPase assays^[6]^. In zebrafish, expression of the ABCB5FL homolog, abcb5, was increased after bile salt injection, and activation of its transcription was associated with decreased bile acid in the liver, suggesting a role for this transporter in the regulation of bile salt secretion^[109,110]^. Regarding ABCB5β, Lutz *et al*. examined the metabolic profile of WT G3361, known to express ABCB5β, or G3361 transfected with ABCB5 shRNA^[111]^. After shRNA, cells had decreased levels of lactate, pyruvate, fumarate, alanine, glycerophosphoethanolamine, and glycerophosphocholine. A small redistribution in the phospholipid pool was also observed. These data suggest a role for ABCB5β in glycolysis. Moreover, because ABCB5β has been shown to mediate progenitor cell fusion, the authors hypothesized that this transporter may be involved in skin tissue turnover and renewal^[2]^.

In atherosclerosis, ABCB5 mRNA expression in microarray was increased in the plaques of high-risk patients compared to low-risk patients^[112]^. Patients were divided into two groups (i.e., high and low risk) based on the ABCD2 prediction tool and CAR score. ABCB5 expression by immunohistochemistry was found in type II macrophages and lymphocytes in the vicinity of neovessels. Therefore, the authors proposed a potential function for ABCB5 in neovascularization^[112]^. A multicenter genome-wide association study (GWAS) showed that ABCB5 SNPs were associated with neurological instability in ischemic stroke, and mouse brain microvascular endothelial cells deprived of oxygen for 3h to mimic cerebral ischemia had an increased number of exosomes, extracellular vesicles secreted by various cell types, expressing ABCB5 protein^[113,114]^. ABCB5 SNPs were associated with changes in the PR interval, i.e., the interval between atrial depolarization and ventricular depolarization in the heartbeat, in another GWAS in the Brazilian population investigating potential genetic variation involved in the development of cardiomyopathy after *Trypanosoma cruzi* infection^[115]^. In a GWAS investigating copy number variants (CNVs), ABCB5 deletion was associated with childhood obesity in African-American and European-American children^[116]^. Whole blood RNA from American adolescents with attention-deficit hyperactivity disorder showed a 1.9-fold increase in ABCB5 mRNA^[117]^. However, all these studies do not present direct evidence, but only correlations, and more research is needed to understand the role of ABCB5 in atherosclerosis, ischemic stroke outcome, cardiomyopathy, childhood obesity, and attention deficit hyperactivity disorder.

Next, Lin *et al*. showed that an ABCB5 SNP (rs2301641) was associated with increased melanin production and decreased transport capacity^[89]^. Furthermore, in the immunohistochemistry of melanoma, cells expressing ABCB5 correlated with non-melanized regions^[51]^. In contrast, ABCB5 expression was not detectable in two amelanotic melanoma cell lines^[3]^. Taken together, this suggests a potential role for this transporter in melanogenesis.

In summary, most of the literature on ABCB5 has focused on its role as a marker of cancer stem cells and its implication in cancer biology and MDR. Few publications have investigated the physiological function of ABCB5FL and ABCB5β. Preliminary data suggest that ABCB5FL is involved in cellular detoxification, while ABCB5β seems to play a role in glycolysis and melanogenesis. However, the results obtained require further investigation and complementary experiments are needed to determine the pathway involved in the observed phenotypes following the knockdown or overexpression of these transporters separately.

No experimental data are available for the soluble isoforms of ABCB5. However, based on their structure, it has been hypothesized that they could function as regulators^[3]^. Of note, ABCB5α is the major isoform expressed in melanoma, and ABCB5.e has been cloned from mouse and human skin^[3,4]^.

## CONCLUSION

In conclusion, a large amount of data on ABCB5 has been generated in recent years. Unfortunately, it remains difficult to disentangle the information proposed in the literature because it is difficult to distinguish between the different isoforms of ABCB5. To date, we know that *ABCB5* encodes for several isoforms, two of which are transporters (i.e., ABCB5β and ABCB5FL) and they have different promoters. ABCB5FL has the typical topology of full transporters and is localized in the testis and prostate. ABCB5β has an atypical topology with a TMD flanked by two NBDs, one of which is truncated at the N-terminus and lacks the Walker A motif and the A-Loop. This transporter has a ubiquitous tissue expression and must homodimerize or heterodimerize to become functional. Both ABCB5β homodimers and ABCB5β/B6 or ABCB5β/B9 heterodimers, identified in melanoma cells, were shown to have basal ATPase activity. Furthermore, ABCB5β, first identified as a marker of skin progenitor cells, was shown to be expressed in a subpopulation of cells with stem cell-like properties in several tissues. This isoform was localized to the endoplasmic reticulum and its potential intracellular trafficking to other organelles requires further investigation. Regarding the involvement of ABCB5FL and ABCB5β in tumorigenesis, a tumor suppressor and oncogene role, respectively, have been proposed. However, since there are conflicting results in the literature and because antibodies do not allow to distinguish between the two isoforms, we suggest adding a rescue experiment with the transfection of ABCB5β and ABCB5FL sequences separately to better identify the transporter responsible for the observed phenotype. With regard to its role in MDR, ABCB5FL was shown to mediate resistance to docetaxel, doxorubicin, and paclitaxel. Other compounds have been proposed to be transported by the two isoforms, but there is no transport assay with separate expression of each isoform to validate these hypotheses. To date, the literature lacks transport assays in vesicles with radiolabeled or fluorescent drugs to determine the extent of the involvement of ABCB5FL and ABCB5β in MDR. Finally, the physiological function of these transporters (ABCB5β, ABCB5β/B6, ABCB5β/B9, and ABCB5 FL) remains unknown and further research may help to better understand their role in normal cells. To this end, we suggest using omics techniques (metabolomics, proteomics, and transcriptomics) to further establish the consequence of ABCB5 isoform downregulation or overexpression in cell lines and animal models. Next, experiments looking at potential substrates, for example, could be performed. Overall, two important points need to be addressed in future publications on ABCB5 (1) to make sure of the isoform studied in a model that does not express both and (2) to validate the anti-ABCB5 antibodies in various applications. Such a strategy will help to generate reliable data to further elucidate the pathophysiological roles of ABCB5 transporters.

## DECLARATIONS

### Copyright

© The Author(s) 2024.
